# Reducing data dimension boosts neural network-based stage-specific malaria detection

**DOI:** 10.1038/s41598-022-19601-x

**Published:** 2022-09-30

**Authors:** Katharina Preißinger, Miklós Kellermayer, Beáta G. Vértessy, István Kézsmárki, János Török

**Affiliations:** 1 Department of Applied Biotechnology and Food Sciences, Budapest University of Technology and Economics, Budapest, 1111 Hungary; 2 Institute of Enzymology, Research Center for Natural Sciences, Budapest, 1111 Hungary; 3 Department of Physics, Budapest University of Technology and Economics, Budapest, 1111 Hungary; 4 Department of Experimental Physics V, University of Augsburg, 86159 Augsburg, Germany; 5 Department of Biophysics and Radiation Biology, Semmelweis University, Budapest, 1111 Hungary; 6 Department of Theoretical Physics, Institute of Physics, Budapest University of Technology and Economics, Budapest, 1111 Hungary; 7 MTA-BME Morphodynamics Research Group, Budapest University of Technology and Economics, Budapest, 1111 Hungary

**Keywords:** Biophysics, Mathematics and computing, Scientific data, Software, Statistics

## Abstract

Although malaria has been known for more than 4 thousand years^1^, it still imposes a global burden with approx. 240 million annual cases^2^. Improvement in diagnostic techniques is a prerequisite for its global elimination. Despite its main limitations, being time-consuming and subjective, light microscopy on Giemsa-stained blood smears is still the gold-standard diagnostic method used worldwide. Autonomous computer assisted recognition of malaria infected red blood cells (RBCs) using neural networks (NNs) has the potential to overcome these deficiencies, if a fast, high-accuracy detection can be achieved using low computational power and limited sets of microscopy images for training the NN. Here, we report on a novel NN-based scheme that is capable of the high-speed classification of RBCs into four categories—healthy ones and three classes of infected ones according to the parasite age—with an accuracy as high as 98%. Importantly, we observe that a smart reduction of data dimension, using characteristic one-dimensional cross-sections of the RBC images, not only speeds up the classification but also significantly improves its performance with respect to the usual two-dimensional NN schemes. Via comparative studies on RBC images recorded by two additional techniques, fluorescence and atomic force microscopy, we demonstrate that our method is universally applicable for different types of microscopy images. This robustness against imaging platform-specific features is crucial for diagnostic applications. Our approach for the reduction of data dimension could be straightforwardly generalised for the classification of different parasites, cells and other types of objects.

## Introduction

Malaria, an infectious disease more ancient than human, causes more than 600 thousand fatalities each year^2^. Due to the interplay of various factors, such as the ongoing COVID-19 pandemic, the disruption of measures against malaria and the global climate change, the number of cases has been increasing again. This circumstance has a large influence on mortality, if the infection is not promptly and effectively treated, which is often the case in endemic regions^3^.

This vector borne disease is caused by five species of the *Plasmodium* genus, where *P. falciparum* is the most widespread and mainly responsible for severe malaria^3^. Following the mosquito bite and the symptom-free liver stage of the infection, the parasites burst out into the blood stream to start their asexual life cycle. During the approx. 48 h of this intra-erythrocytic cycle, they mature through the ring, trophozoite and schizont forms. At the end of the cycle, they multiply, and the merozoites begin the next cycle by invading new RBCs^4^. The intra-erythrocytic cycle has been the subject of intense research because it causes the main clinical symptoms and is the major target of diagnostics and antimalarial treatment^4,5^.

Nowadays, the gold-standard diagnostic tool is still light microscopy on Giemsa-stained smears, which relies on human performance both in terms of sample preparation and visual inspections, making this method time-consuming and subjective^6^. The persistence of microscopy that is rooted back into its long worldwide tradition and the general concept of *seeing is believing*, defers the spread of other methods. Currently, rapid diagnostic tests represent the most competitive alternative of light microscopy due to their easy use and affordable price. However, they are not quantitative and impose a compromise on sensitivity and species specificity^1,7^. There have also been other methods like Polymerase-Chain-Reaction (PCR)^8^, flow cytometry^8^ and magneto-optical detection^9,10^ developed for malaria diagnosis, however, currently they are not available in a point-of-care format required for high-throughput in-field diagnostics.

During maturation, the parasites change the topography^11–14^ and the optical properties of the host RBCs^15^. In fact, it is well-established that malaria-infected RBCs can be characterised by their morphological properties that can also be correlated with their characteristic fluorescence patterns^16,17^. These new insights and the widespread use of microscopy, triggered new approaches for the automatised recognition of malaria infected RBCs in blood smears, which can lead to a breakthrough in malaria diagnosis. These efforts are well exemplified by recent works reporting on NN-based algorithms for the recognition of infected RBCs^18–27^, which can even run on standard cell phones. While these pioneering studies clearly demonstrate the potential of NNs for automatised malaria diagnosis, this approach still requires substantial improvement in terms of sensitivity, specificity to different malaria species and stages, as well as robustness against the imperfectness of microscopy images. The other challenge is to improve the performance while keeping the computational costs low. In the following, we discuss the state-of-the-art in this field through a few selected studies.

As an important new direction, it has been shown recently that NNs do not only work on images of Giemsa-stained blood smears but also perform well on label-free light microscopy images^17^. This also gives the hint that malaria diagnosis can benefit from the advantages of NNs in analysing images recorded by microscopy methods other than light microscopy. In fact, recently there is an increasing number of techniques successfully applied for high-resolution imaging of malaria infected RBCs, including topographic imaging by atomic force microscopy (AFM)^13,14,16^ and infrared nano-imaging^12^.

The main goal of NN-based algorithms developed for malaria infected RBC recognition in light microscopy images is to increase the throughput of diagnosis by reducing the image processing time and to enhance its reliability by eliminating human errors^28^. The most efficient approaches typically apply a two-step scheme, where healthy and infected RBCs are distinguished in the first step, followed by a stage-specific classification of infected RBCs in the second step^29–32^. Most of the efforts have been focused so far on Giemsa-stained images, where several attributes of the infected RBCs have been successfully exploited in the recognition process, including the characteristics of the colour scheme^30^, the morphology of the RBCs^31^ and their other statistical features^32^. The accuracy of these methods ranges between $\sim$ 80–98%. Similar performance levels have recently been achieved also by unbiased convolutional networks with minimal or no pre-processing of microscopy images^33–36^.

All the above mentioned imaging techniques have two important limitations for NN-based recognition. They can provide limited training sets with typically a few thousands images, where healthy RBCs and RBCs containing the different stages are rather unevenly represented. Even when working with parasite-enriched cultures^37–39^, the number of uninfected RBC images is approx. 10 times larger than that of the infected ones. Such imbalance in the data set can result in the so-called overfitting of the images^40^, lowering the accuracy of the NN-based recognition. As a common solution for this problem, data augmentation can be used to equalise the number of images in the different categories (ring-, trophozoite-, schizont-stage, and healthy RBCs) by generating more data for the training set^33,34^. This has been demonstrated to improve the performance of the NN^35,36^ and to further increase the generalisation ability.

Even after the data augmentation process, the image set is often too small to properly train a two-dimensional convolutional NN for the stage-specific classification of RBCs due to the large number of parameters to be fitted by the NN. If the neural network cannot be further optimized then the only possible way to reduce the number of trainable parameters is to decrease the size of the input. This can be done either by reducing the resolution of the images^27^ or by reducing the dimension of the input by selecting the characteristic features of the data^41^. This latter approach comes with an enormous reduction of the input data with often increasing efficiency^31,32,42^.

Here, we developed a novel NN-based approach for the full classification of RBCs into four categories: healthy, infected by ring-, trophozoite-, and schizont-stage malaria parasites. Most importantly, our method is not optimised for inputs from a certain type of imaging technique but can handle two-dimensional RBC images recorded by fundamentally different techniques. The general applicability of the method is demonstrated via the high-accuracy four-category classification of RBCs using Giemsa-stained light microscopy images, AFM images and fluorescence microscopy images. We also show that the smart reduction of image dimension, achieved by selecting characteristic RBC cross-sections, does not only speed up the algorithm but also boosts its accuracy, which is found to be > 95% for each of the four types of image sets. Beyond the efficient recognition and classification of other parasites and cells, our approach could be generalised straightforwardly for the analysis of general objects with characteristic cuts.

## Results

### Main steps of the approach

Our aim is to create an efficient NN-based method, which can process data obtained by arbitrary imaging methods and is able to sort RBCs into the four aforementioned categories. In addition, we require this algorithm to have a performance of > 95%, even in case of limited training sets and low computational capacity. In the following, we outline the main steps of our study: (i) imaging, (ii) cell identification, (iii) data augmentation, (iv) reduction of image dimension, and (v) classification.

**Figure 1:**
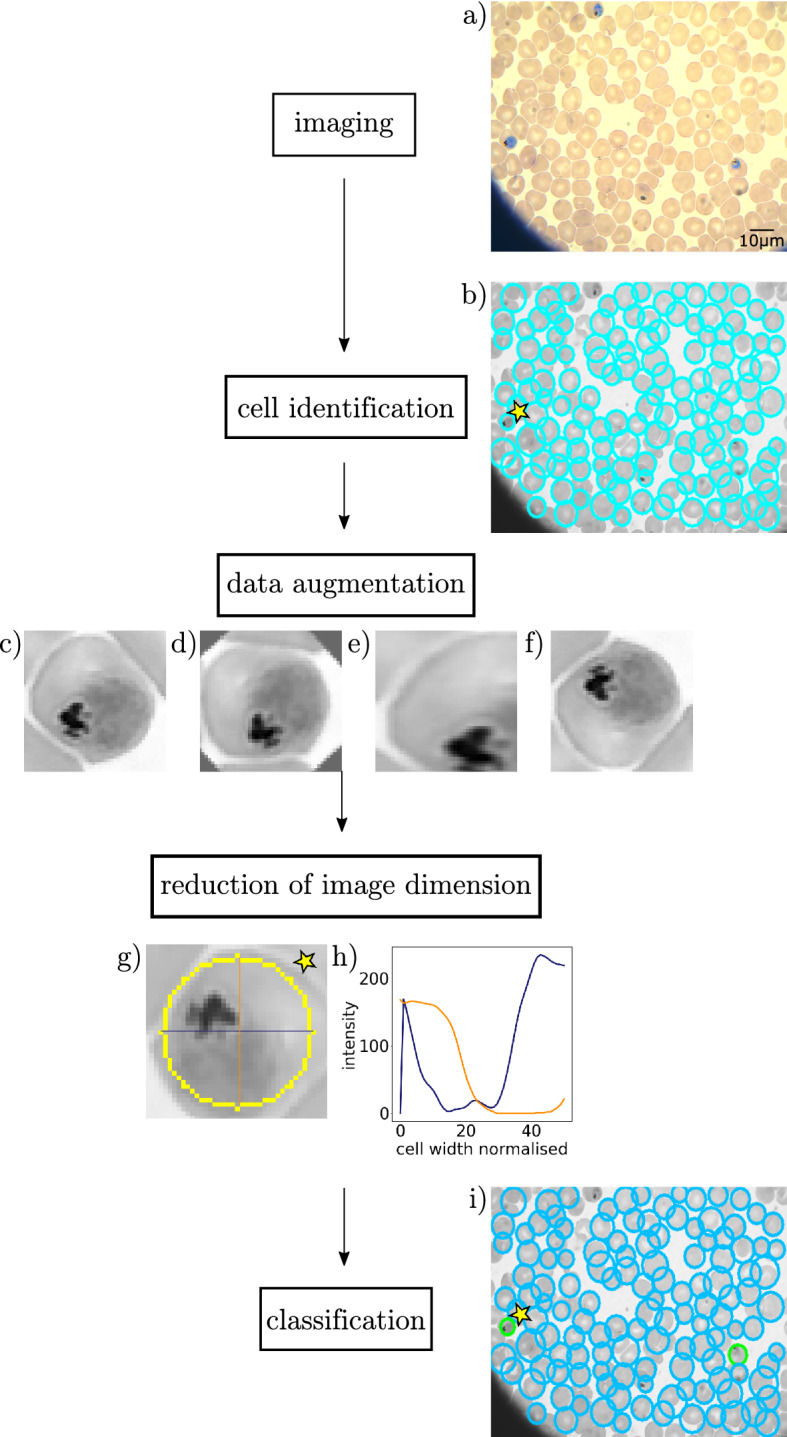
Schematics of the workflow for the stage-specific detection of malaria-infected RBCs. (**a**) Imaging: raw light microscopy image. (**b**) Cell identification: the detected cells are marked by cyan circles. (**c**,**d**) Data augmentation: the original image (**c**) is rotated (**d**), scaled (**e**), and reflected (**f**). (**g**,**h**) Reduction of image dimension: the most asymmetric cross-sections are extracted from the cell image. (**i**) Classification: the identified cells are classified by a neural network.

(i) All types of image sets were recorded by our group in our study, except for Giemsa-stained light microscopy data, which were taken from Abbas et al.^43^ This data set contains images from 17 malaria-infected patients (Fig. 1a).
(ii) To identify nearly circular RBCs, we used the Hough gradient method^44^, which is less sensitive to the type of the imaging method and the corresponding contrast channel as compared to thresholding (Fig. 1b).
(iii) In order to train the NN efficiently for the categorisation of RBCs, we used data augmentation in the training data set to equalise the number of available images in each category (healthy, ring, trophozoite, and schizont) and to increase the training set (Fig. 1c–f).
(iv) The classification by convolutional NNs requires the fitting of thousands of weights, however, the number of images available for training was rather limited, approx. 90,000 microscopy images, even after the data augmentation process. We overcame this challenge by drastically reducing the number of fitting parameters via the reduction of image dimension in the following systematic way. We extracted the most asymmetric cross-section for each RBC, usually crossing the parasite, in approx 50–60% of the cases for ring-stage parasites and in more than 85% of the cases for trophozoite-stage parasites (Table 3), and three more cross-sections, at $\pm 45^\circ$ and 90$^\circ$ with respect to the asymmetric cut. The schizont-stage parasite is not included in the statistics because the cut always captures the parasite due to its size. Since the cylindrical symmetry of RBCs may be reduced not only by the parasites but also because of the environment of the cells, we weighted down the periphery of the RBCs using a Gaussian filter, before extracting the four cuts (Fig. 1g,h).
(v) Finally, we applied a one-dimensional convolutional NN for the full classification of RBCs in three ways, using only the asymmetric cross-sections, the asymmetric cross-sections combined with the 90$^\circ$ cut, and all the four cross-sections. The performance was the best in the latter two cases, significantly better than the performance of the two-dimensional convolutional NN applied to full images (Fig. 1i).

In the following sections, we describe these steps in detail.

### RBC imaging techniques

**Figure 2:**
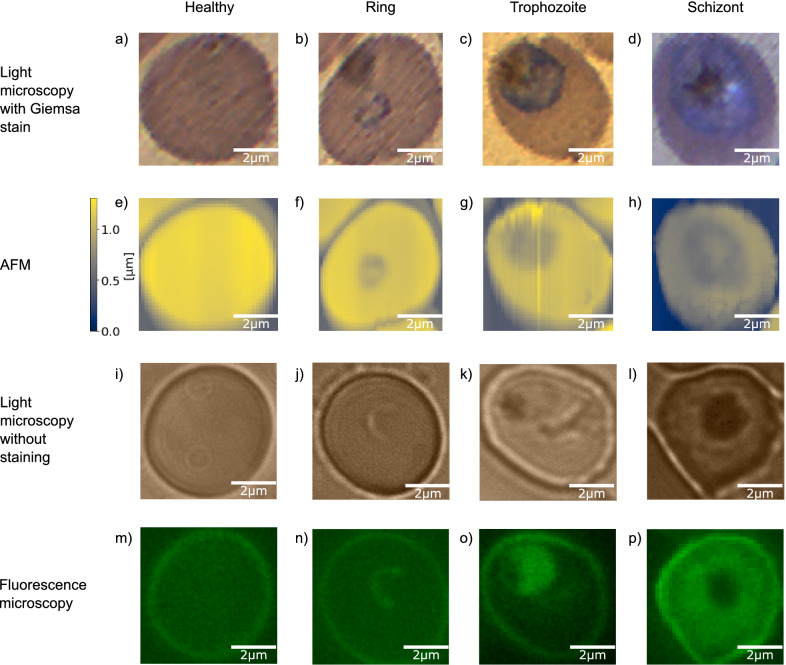
Representative microscopy images of malaria blood stages: healthy, ring, trophozoite, and schizont stage red blood cells are shown in columns respectively from left to right. Images in the different rows are recorded, using different imaging techniques. (**a**–**d**) Light microscopy images of Giemsa-stained RBCs. (**e**–**h**) Atomic force microscopy was used to obtain topography images on the same RBCs as in the row above. The colour bar left of (**e**) indicates the height scale common for all topography images. (**i**–**l**) Unstained light microscopy images, showing a different set of RBCs. (**m**–**p**) Fluorescence maps of the same RBCs as in the row above. The fluorescence signal was measured after excitation at 405 nm without using dyes.

The input images for our NN were recorded with three fundamentally different imaging techniques: light microscopy of Giemsa-stained smears, AFM, and fluorescence microscopy. While it is the most commonly used technique, conventional imaging of infected RBCs by light microscopy requires the use of dyes. In contrast, AFM is a label-free imaging technique, capturing the morphological properties of the RBCs and revealing features characteristic to each parasite stage. Similarly, fluorescence maps were recorded without dyes by taking advantage of the auto-fluorescence of infected RBCs excited by light of 405 nm wavelength.

All these methods can image areas considerably larger than individual RBCs. In our studies, there are typically a few dozen of RBCs covered in a single image. In Fig. 2 we show exemplary single-RBC cutouts for all the imaging techniques (rows) and all the four cell categories (columns). The first and second rows show images of the same set of RBCs obtained by Giemsa-stained light microscopy and AFM, respectively. The third and fourth rows show images of another set of RBCs, recorded by using label-free light and fluorescence microscopy, respectively. The four columns of Fig. 2 correspond to healthy RBCs and RBCs infected by ring-, trophozoite-, and schizont-stage parasites.

For segmentation into single-RBC cutouts that were eventually used for the NN-based four-category classification, we used 173 AFM images, 1232 fluorescence microscopy images and 792 Giemsa-stained light microscopy images [747 from Ref.^43^ and 45 recorded by us]. Label-free light microscopy images were taken in a limited number to help an unambiguous stage-specific labelling of RBCs in the corresponding fluorescence images and were not used in the NN analysis.

### Segmenting and labelling images

For image processing, we made a cell detection program, which automatically detects individual RBCs in any kind of microscopy images used in our study and segments them into single-RBC cutouts. These single-RBC images were then classified and sorted into the four aforementioned categories by three experts.

**Figure 3:**
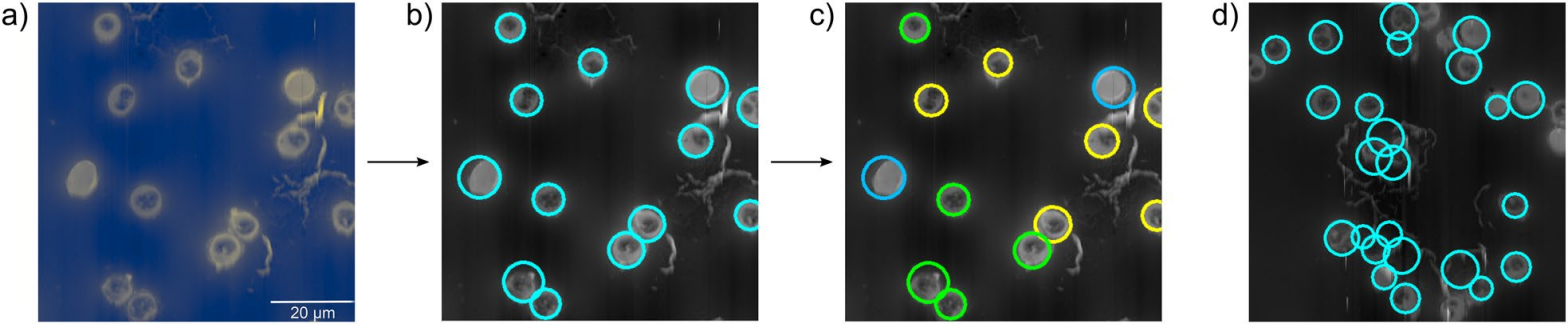
Illustration of the extraction process of single-RBC images from AFM topography pictures. (**a**) Raw topography image. (**b**) Cell identification: individual RBCs are detected by the Hough gradient method and marked by cyan circles. (**c**) Manual classification: the cells are labelled by experts as healthy (lightblue), infected by ring-stage (blue), trophozoite-stage (yellow) and schizont-stage (green) parasites. (RBCs infected by rings are not present in the sample image.) Cell extraction: The detected cells are extracted from the raw image and saved with the respective label. (**d**) Sample image of undetected RBCs and false identifications.

To process a raw image by this program, at first the user has to select the microscopy method used for the image acquisition [see Fig. 3a]. In the second step, the cells in the image are detected by the Hough gradient method^44,45^. The RBCs are mostly circular or at least a large enough section of their perimeter is circular so that this gradient based method can identify them with high accuracy, as depicted in Fig. 3b. For high-efficiency RBC recognition, the sensitivity of the edge detection and a range for the radius were predefined. With this method, we were able to find more than 95% of the cells in all microscopy images, which slightly varies with the imaging technique (Table 1), with a negligible amount of false detection (for more details see “Methods” section: “Preprocessing of the microscopy images”). While most of the cells can be found with our method, it fails for cells strongly deviating from a circular shape. As a result, we obtained centre position and radius values for each RBC.

Following the segmentation, three experts manually classified the single-RBC images as healthy, infected by ring-, trophozoite-, schizont-stage parasites, as indicated by circles of specific colours in Fig. 3c. The single-RBC images were then saved with labels, corresponding to their category. The experts had the possibility to label wrong identifications by “no RBC” (Fig. 3d). The few images marked with this label were dropped and excluded from any further analysis. These labelled single-RBC images were used as inputs for the NN analysis.

The Hough gradient method returns the centre position and a radius of the detected RBC. The square, containing this circle, is cut out from the image and used as the input image for the convolutional neural network.

After pre-processing and labelling, we obtained a data set of 26223 single-RBC images for light microscopy (24648 from^43^ and 1575 recorded by us), 7386 images for AFM, and 45726 images for fluorescence microscopy. Table 2 shows the distribution of the recorded RBCs over the different categories for each imaging method. As clear from these numbers, although we used cultures with high parasitemia for sample preparation, the number of healthy cells was much larger for each microscopy technique than the number of infected cells, leading to an imbalance between the categories.

**Table 1:** Probability of cell detection for light microscopy, AFM, and fluorescence images.

Imaging method	Detection probability
Light microscopy	95%
AFM	96%
Fluorescence microscopy	97%

### Performance of the NN on imbalanced and balanced data sets of 2D RBC images

At first, we apply our convolutional NN for the four-category classification of RBCs using 2D microscopy images. Our main aim here is to demonstrate the need for data augmentation. Furthermore, the performance of our convolutional NN on the augmented sets of 2D images will serve as a standard to demonstrate that the performance of this convolutional NN can be boosted by using aptly chosen 1D cuts of the single-RBC images as inputs, instead of the whole images.

**Table 2:** Distribution of healthy RBCs, RBCs infected by the different malaria stages in the whole set of single-RBC images. The corresponding numbers are shown for all imaging methods used in the NN-based recognition. In addition, numbers for the “no RBC” images are also included.

Imaging method	Total	Healthy RBC	Ring	Trophozoite	Schizont	No RBC
Light microscopy	26,223	24,586	502	575	560	1662
AFM	7386	5679	350	558	799	525
Fluorescence microscopy	45,726	40,874	1946	1598	1308	1265

For a proper comparison, we decided to use the same NN with four convolutional and one hidden dense layers for all three imaging methods and for the analysis of both 2D images and their 1D cuts (for details see the “Materials and methods” section). This allows us to study the dependence of the performance solely on the supplied input data, while the scheme of the NN is kept unchanged. For efficiency tests, we always used a random 90–10% train-test data set split.

**Figure 4:**
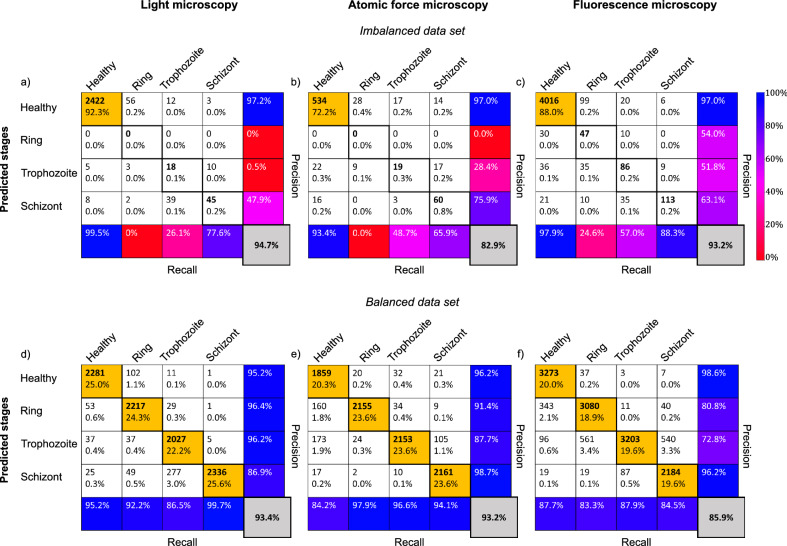
Classification performance of the convolutional NN on 2D microscopy images. The performance parameters, as obtained on imbalanced (non-augmented) sets of light microscopy, AFM and fluorescence microscopy images, are respectively summarised in the confusion matrices of panels (**a**), (**b**), and (**c**). The confusion matrices obtained for the corresponding balanced (augmented) data sets are shown in panels (**d**), (**e**), and (**f**). The cells in the single-RBC cutouts were classified into four categories: healthy, ring, trophozoite, and schizont. The confusion matrices show the performance of the classification for each microscopy technique on the test set. The labels of the rows are the categories predicted by the NN-based classifier, while the labels of the columns indicate the classification by human experts. Correspondingly, the sum over a given column/row gives the number of cases falling into that category by human/NN classification. The diagonal elements correspond to the number of RBCs correctly classified by the NN, while the off-diagonal elements show false classifications. In each field, the number of counts as well as the corresponding percentage with respect to the total set are shown. In the last column, the precision for each of the four categories is shown. In addition, the recall values are displayed in the bottom column. Most importantly, the overall classification accuracy is displayed in the grey field in the bottom right corner. For the definition of the precision, recall, and overall accuracy values see the main text. For visual guidance, the diagonal fields with percentages > 18% have an orange background, while fields with the precision values (last column) have background colors according to the common colourbar on the right.

First, we tested the NN on the original data sets with strong imbalance in the categories. The results are summarised in the tables in Fig. 4a–c. Precision defines the ratio of correctly classified results and all results classified as the respective row, while the recall represents the ratio of correctly classified results and all results belonging to the respective column. The overall accuracy (grey box) is then calculated as the ratio of correctly classified cells, independent of the category, and all classified cells. Its score of 83–95% is not bad but this is solely the result of the extremely high success rate of the healthy cells, highlighted by blue in the top-left corner of the tables. As a known consequence of a strongly imbalanced set, when 80–90% of the data belongs to one category, it is extremely hard to teach a network to achieve a success rate well beyond the percentage of data belonging to the largest category. Thus, in our case, this would lead to the NN predicting always a healthy RBC.

Therefore, we had implemented a data augmentation method to balance the data set (see “Materials and methods” section for details). The augmented images of RBCs are automatically labelled to be the same as the original non-augmented image. In Fig. 4d–f, we show the confusion matrices of our tests using the same NN but a balanced data set. The results are largely improved with respect to the original non-augmented data set. Except for the prediction of trophozoite stages in fluorescence microscopy images, the precision of the prediction (last column of the confusion matrix) is above, typically well above, 80% for all four RBC categories and for all three imaging techniques. Overall, a high classification accuracy was achieved for all microscopy techniques, 93.4% for light microscopy, 93.2% for AFM and 85.9% for fluorescence microscopy. This performance level is similar to that achieved, e.g., in Ref.^36^.

A deeper dive into the precision of the predicted stages on the augmented data sets shows that early stages of malaria, i.e. rings and trophozoites, are best recognised in Giemsa-stained light microscopy images, while the schizont stage is captured most in AFM and fluorescence microscopy images.

### Performance of the NN on characteristic cuts of single-RBC images

To reduce the computation time, the large number of parameters to be fitted by the NN, and, in turn, the probability of overfitting, we decreased the amount of input data by selecting characteristic cross-sections of single-RBC images. In other words, we reduce the dimension of the input data by supplying the NN with 1D instead of 2D data. In this step, we have to optimally select cross-sections of the RBCs, which capture best the features associated with the presence/absence and the life stage of the parasites. In this respect, there are various possible choices of measures or indicators, for instance, the sum of local changes over the whole RBC or its asymmetry. The former is very sensitive to external noise, which is often present in AFM and fluorescence images, e.g., due to contamination of the AFM tip^46^ (see Figs. 2g and 8). Therefore, we opted for the second method and quantified the overall asymmetry of the RBCs to avoid local noise and layering problems.

**Figure 5:**
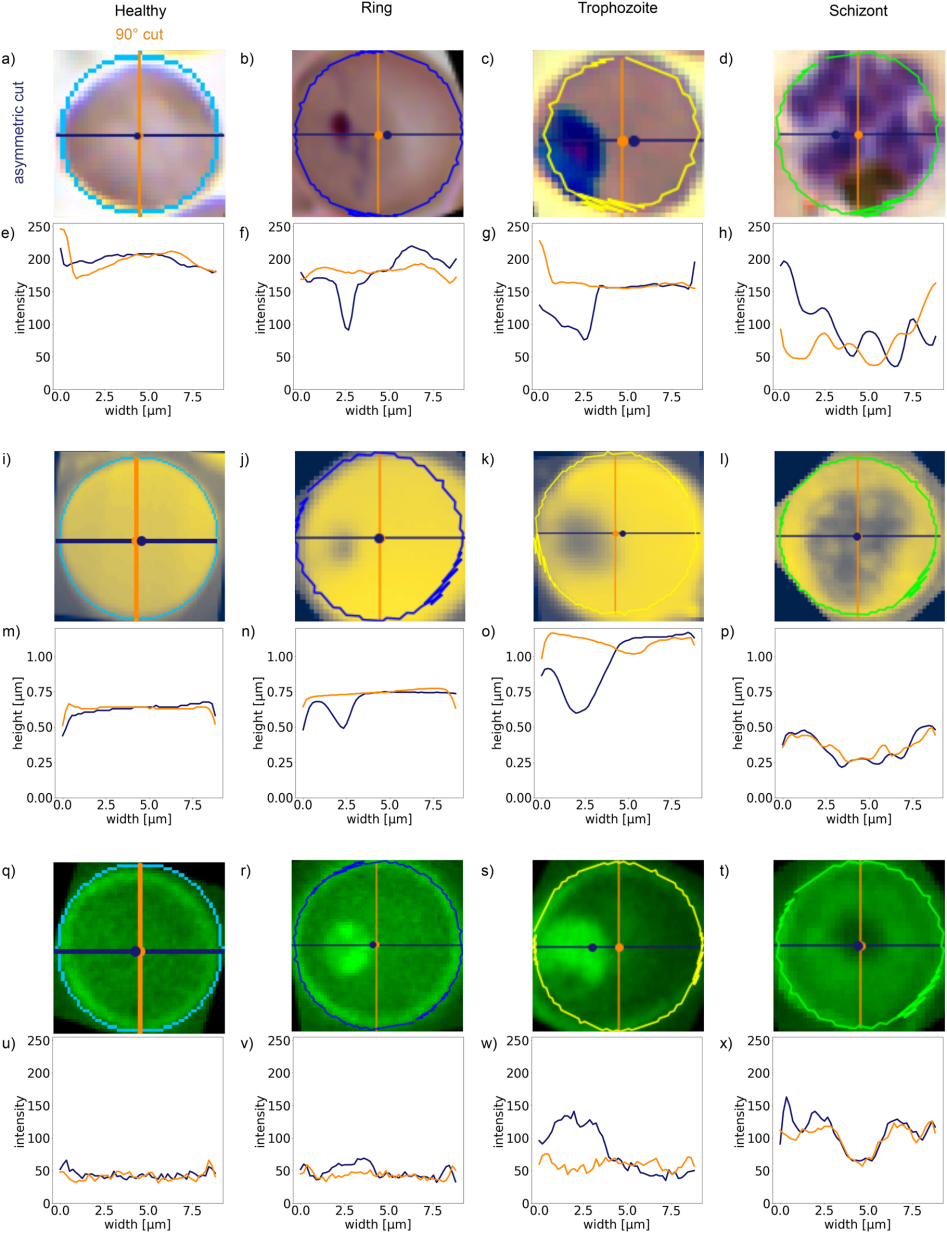
Characteristic cross-sections through single-RBC images. Similar to Fig. 2, columns show the four different categories (healthy RBC and RBCs infected by ring, trophozoite and schizont stages), while rows correspond to different imaging methods. (**a**–**d**) Light microscopy images of Giemsa-stained RBCs. The coloured circles surrounding the detected cells are the determined cell contours (see also Fig. 3). The geometric centre and the centre of mass are marked by an orange and a blue dot, respectively. The asymmetric cut connecting the two centres and the 90$^\circ$ cut only crossing the geometrical centre are indicated by a blue and an orange line, respectively. (**e**–**h**) Intensity profile for the asymmetric (blue) and the 90$^\circ$ (orange) cuts in the images above. (**i**–**l**) AFM images of RBCs and (**m**–**p**) their corresponding height profiles along the two cuts. (**q**–**t**) Fluorescence images of RBCs and (**u**–**x**) their corresponding intensity profiles along the two cuts.

Our method to quantify the global asymmetry of the RBCs is illustrated in Fig. 5. The perimeter of the cell is determined by the circle fit of the contour. We calculate the geometric centre $r_{\text {geo}}$ and centre of “mass” $r_{\text {mass}}$ of an RBC using the conventional definitions:

1 $$\begin{aligned} r_{\text {geo}}&=\frac{1}{N}\sum _{i,j}r_{ij} \end{aligned}$$

2 $$\begin{aligned} r_{\text {mass}}&=\frac{1}{N}\sum _{i,j}h(r_{ij})r_{ij}, \end{aligned}$$

where $r(_{i,j})$ denotes the position of the pixel with the *x* and *y* coordinates *i* and *j*, respectively, and the summation goes over all *N* pixels within the interior of the RBC. In the second sum, $h(r_{ij})$ denotes the height (AFM) or intensity (light and fluorescence microscopy) of a pixel at $r_{i,j}$.

We define the asymmetric cut as the cross-section going through both centres. In Fig. 5, the geometric centre is indicated with an orange dot and the centre of mass with a blue dot in each image. The blue line corresponds to the asymmetric cut connecting them. The two centres are generally close to each other for healthy RBCs (first column), as they are nearly cylindrical and featureless. In infected RBCs, especially in later stages, the asymmetric line typically goes through the parasite. This is because the parasites, distinguished by their optical and mechanical properties, introduce strong features in the intensity and height profiles in the light (fluorescence) microscopy and AFM images of the RBCs, respectively, hence they affect the position of $r_{\text {mass}}$. For off-centred parasites $r_{\text {mass}}$ departs from $r_{\text {geo}}$, while for parasites close to $r_{\text {geo}}$ the two centres are nearly identical. Importantly, in both cases the asymmetric cut is expected to go through the parasite.

With dimension reduction, it is easy to go too far and the reduced amount of data may not contain enough information to carry out reliable categorisation. Therefore, we applied data reduction in multiple subsequent steps. Following the NN-based analysis of 2D single-RBC images, described in the previous section, we used four distinct 1D cuts as inputs, namely the asymmetric cut and three further cuts spanning 90$^\circ$ and ± 45$^\circ$ with it. As the next step, only the asymmetric and the 90$^o$ cuts were used as inputs. Finally, the NN-based categorisation was carried out, using only the asymmetric cut. Correspondingly, the number of input pixels were reduced from $n^2$ to 4*n*, *n*, and finally *n*.

We compare the performance of the NN in the four cases later in the discussion section. Since the performance was the best for the asymmetric cut combined with the 90$^\circ$ cut (2*n* pixels), this part of the work is described here in detail.

Even rows in Fig. 5 show the intensity profiles of RBCs for the asymmetric (blue) and the 90$^\circ$ cuts (orange). We observe similar features for all three experimental methods. The cuts of the healthy RBCs, displayed in the first column of Fig. 5, show predominantly flat profiles for both cuts, which are very close to each other, reflecting the cylindrical symmetry of the healthy RBCs. We believe that these features are the key why the NN performs better with these two cuts as compared to the single (asymmetric) cut input. On one hand, the presence of a parasite can be excluded with a high probability, if both the asymmetric and 90$^\circ$ cuts are flat and nearly identical. On the other hand, systematic errors of the imaging process can be cancelled by the difference of these two profiles, as seen, e.g., in Fig. 5e. Importantly, as the parasites matures, the infected RBCs develop more and more features with variances reaching the intensity levels. The schizont-stage parasites typically fill up most of the RBC, thus cuts going through the geometric centre always intersect the parasites.

**Table 3:** Success rate of asymmetric cuts going through the parasite.

Imaging method	Ring	Trophozoite	Schizont
Light microscopy	51%	88%	100%
AFM	59%	86%	100%
Fluorescence microscopy	88%	95%	100%

**Figure 6:**
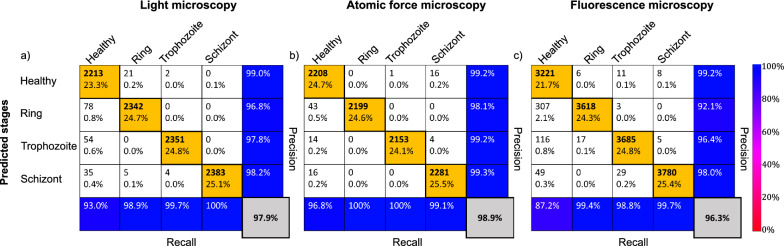
Classification performance of the convolutional NN on two orthogonal 1D cross-sections. The performance parameters for light microscopy, AFM and fluorescence microscopy, as obtained using two perpendicular cuts of single-RBC images, are respectively summarised in the confusion matrices of panels (**a**), (**b**) and (**c**). For the description of these perpendicular cross-sections, the asymmetric and the 90$^\circ$ cuts, see the main text. Notations, labels and colours follow the same convention as in Fig. 4. For the classification here, the balanced (augmented) image sets were used, as in Fig. 4d–f. The overall classification accuracy is highly improved with respect to the classification using the 2D images and reaches more than 96% for each microscopy technique: 97.9% for light microscopy, 98.9% for AFM, and 96.3% for fluorescence microscopy.

Prior to the NN analysis we quantified how often the asymmetric cut locates the parasite in single-RBC light microscopy, AFM and fluorescence microscopy images, by visually checking if this cut goes through the parasite. Table 3 shows the percentage of asymmetric cuts going through parasites for RBCs infected by ring- and trophozoite-stage parasites. In light microscopy and AFM, the asymmetric cut captures the ring-stage parasite in approx. 50–60% of the cases, while it shows a hit rate of 88% in fluorescence images. For trophozoite-stage parasites, the rate is much higher with more than 85% for light microscopy and AFM and 95% for fluorescence microscopy images. The rate was not determined for the schizont stage, as these parasites are typically extend over a large fraction of the cell, hence they are necessarily crossed by the asymmetric cut. The success rate of the asymmetric cut locating the parasite strongly depends on the contrast of the images. Edge effects like overlapping cells or misaligned contours, capturing the background of the image, play a large role in low contrast images, which is the case for most of the ring-stage parasites in AFM and light microscopy. In spite of all these troubles the NNs surpass the above success rates.

As the next and perhaps most important step towards the validation of our new approach, we provided the two characteristic cuts as input to our NN-based classifier. As shown in Fig. 6a, the overall accuracy values reached 97.3% on the light microscopy images, 98.9% on the AFM images (Fig. 6b), and 96.3% on the fluorescence microscopy images (Fig. 6c). The equally high performance for each imaging method demonstrates the general applicability of our approach for different types of imaging techniques. The method also worked uniformly well for all stages, except for the ring-stage parasite of the fluorescence microscopy, which had the aforementioned contrast problem. The limitations discussed for the success rate of the asymmetric cut influence the performance of the NN, as it gets the most characteristic features from this step. While incorrectly classified cells mostly originate from this issue, the NN still outperforms the success rate, as the relation between both cuts also contributes to the classification.

**Figure 7:**
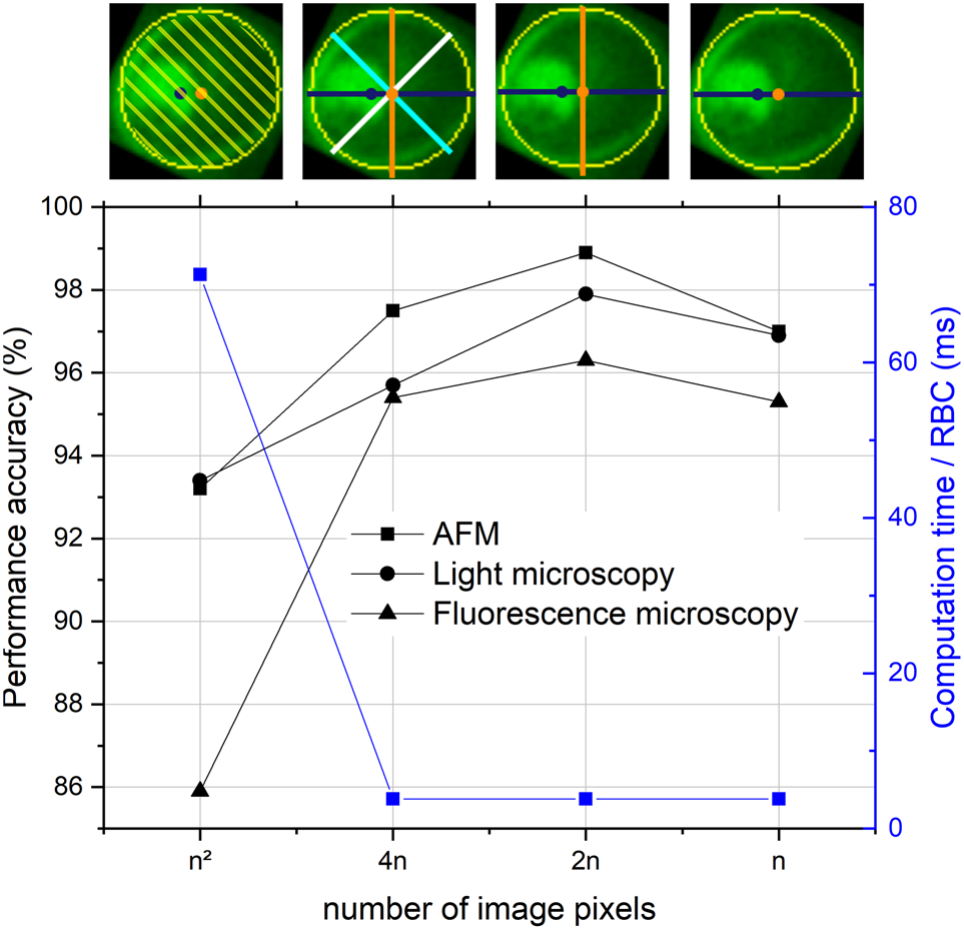
Classification performance of the convolutional NN and computation time per RBC versus the amount of input data for light microscopy, AFM, and fluorescence microscopy images. The NN was trained and tested on 2D RBC images ($n^2$ pixels), four cross-sections (4*n*), two characteristic cross-sections (2*n*), and one single cross-section (*n*), the asymmetric cut. These four cases are illustrated by exemplary fluorescence images on the top. Asymmetric cuts are indicated with blue lines, while the 90$^\circ$, $+$ 45$^\circ$ and − 45$^\circ$ cuts are shown in orange, cyan and white, respectively.

Importantly, the smart reduction of the input data significantly improved the performance of the NN with respect to the classification using the 2D images, as clear from Fig. 7. At the same time, the data reduction lowered the computation time (training duration per epoch) by one order of magnitude, namely from 71.3 ms per RBC for the 2D images to 3.8 ms for the two characteristic cuts on a standard laptop, running with windows 10, x64-based processor, and a processor speed of 2.50 GHz. While the calculation of the centre of mass requires 115 ms per RBC, the overall computation time for 2D images already exceeds this additional time span by 12%, when training for more than one epoch.

## Discussion

In this work, we introduced a NN-based approach for the classification of RBCs into four categories: healthy, infected by ring-, trophozoite- and schizont-stage malaria parasites. We created a universal method for cell-contour detection, data handling and stage-specific recognition, which is not limited to a certain type of imaging technique. The universality of the approach has been demonstrated on sets of RBC images recorded by light microscopy, AFM and fluorescence microscopy.

The main challenges for the high-performance stage-specific classification of RBCs using NN algorithms are the limited number of 2D microscopy images typically available for training and the uneven distribution of healthy and infected cells. The former can compromise the performance of convolutional NNs due to the large number of parameters to be fitted and the latter makes NN also susceptible to overfitting. We show that increasing the training set, which does not require additional manual labelling, and making the distribution of the RBC categories balanced via data augmentation partially solves these problems, irrespective of the type of microscopy images. This step leads to an overall accuracy of the NN-based prediction ranging between 86–93% for the three types of imaging techniques tested here that is comparable to performance values reported recently for NN-based malaria detection^33–36^.

The key point of our approach is the reduction of data dimension by a careful selection of features, which leads to a remarkable increase in the performance of the NN-based classification compared to the 2D images. This reduction is achieved by the automatised determination of characteristic cross-sections through the RBCs. We introduce the most asymmetric cut of the RBC that connects its centre of mass with its geometrical centre and intersects the parasite in most cases. The asymmetry of the RBC is a good global measure, insensitive to noise in the images, and the asymmetric cross-section captures the most characteristic features of the infected RBC. Combining this cut with a perpendicular cross-section going through the geometrical centre also helps to eliminate systematic errors and artefacts of imaging. Using these two cuts as the input of the NN-based classifier, we provide a reliable tool for automatised stage-specific recognition of malaria infected RBCs.

In order to determine the optimal degree of data reduction for the NN-based classification, we gradually reduced the size of the input data from $n^2$ pixels (2D images) to 4*n*, 2*n*, and finally *n* pixels. The last corresponds to the case when only the asymmetric cut was used as input data, while in case of 2*n* and 4*n* it was combined with the 90$^o$ cut and also with the ± 45$^\circ$ cuts, respectively. Figure 7 shows the overall accuracy of the malaria stage detection versus the amount of input data supplied to the convolutional NN for all three imaging methods.

The asymmetric cut alone already outperforms the two-dimensional images, but by including other cuts, the recognition gets even better. The optimum is achieved at 2*n*, when the asymmetric cut is combined with the 90$^\circ$ cross-section. Our conjecture is that this helps the system to work on a differential basis and not be affected by the actual values of the intensity. As another important effect discernible in Fig. 7, the reduction of dimension not only improves the classification but also reduces the computation time per RBC by a factor of 18 to 3.8 ms.

Although we used smears made from cultures of high parasitemia for recording microscopy images, the number of infected RBCs was still considerably lower than that of the healthy ones. To equalize the number of RBCs in the four categories, we used data augmentation, which turned out to be an essential step for the training of the network, as described in the manuscript. However, once the neural network is trained on such a balanced set of the four categories of RBCs, the accuracy of our classification, that is $\approx$ 97–99% for all 4 categories in case of light microscopy images, does not depend on whether the new sample has low or high parasitemia, since this number tells the accuracy of individual RBCs being classified properly.

With our novel, high-accuracy classification approach of RBC images, which is applicable to fundamentally different types of imaging techniques, we make a contribution to meeting the rising need for reliable and fast malaria diagnosis. In fact, the universality and the robustness of our method against imaging platform-specific features naturally grants its applicability for the wide range of light microscopes used worldwide for malaria diagnosis that is a prerequisite for successful in-field applications. Our NN-based method could be generalised for various other applications, such as the recognition and classification of other parasites, cells or any type of objects possessing characteristic cuts. The smart reduction of data dimension, a key concept in our approach, is expected to boost the accuracy of NN-based classification when the training set is limited, as is typically the case for microscopy images.

## Methods

### Sample preparation

Cultures of *P. falciparum* parasites from the laboratory adapted strain 3D7 were maintained in culture medium (Albumax, 25 mg/L Gentamycin, RPMI 1640) in an atmosphere of 5% $\hbox {CO}_{{2}}$ and 5% $\hbox {O}_{{2}}$ as in Ref.^47,48^. The cultures were raised to > 5% parasitemia for each measurement and used to make a thin film smear on VWR microscope slides (90 ground edges, nominal thickness 0.8–1.0 mm).

### Morphological measurements

**Figure 8:**
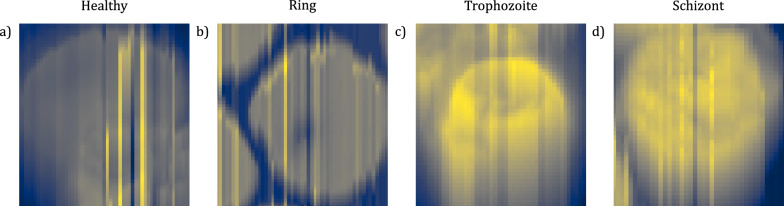
Artefacts in AFM images due to tip contamination. (**a**–**d**) Representative images for each cell category. All images were classified correctly by the neural network, showing its insensitivity to local variations in height.

To measure the morphology of RBCs on dried smears, we used an MFP-3D AFM (Asylum Research, Oxford Instruments) as in Ref.^16^, which was operated in AC mode at a scanning speed of 0.25 Hz. Each scan was performed on an area of 90 $\times$ 90 $\upmu$m with a resolution of 512 $\times$ 512 pixels. The cantilever was an OTESPA-R3 from Bruker with a rectangular shape, a tip radius of 7–10 nm, a spring constant of 26 N/m, operated at a frequency of 280–300 kHz with a drive amplitude of 250–300  mV. The software used for the set up and acquisition of the AFM was Igor Pro 6.37. Fig. 8 shows representative images of RBCs with artefacts due to tip contamination. Despite the bad quality of the image, these cells were classified correctly.

### Fluorescence measurements

The fluorescence microscopy images were obtained as in Ref.^16^ by using an Olympus IX81 inverted microscope, operated at a wavelength of 405 nm. Throughout the experiments, we used an Olympus TIRF objective (UApo N, 100x, 1.49NA). Our samples were excited by an OMICRON (Rodgau-Dudenhofe, Germany) LaserHub, which contained four independent laser sources (405 nm 120 mW CW diode, 488 nm 200 mW CW diode, 561 nm 156 mW CW diode, 642 nm 140 mW CW diode). The lasers were operated at 5–20% power controlled by the OMICRON Control Center software (v.3.3.19). An EM-CCD camera (Andor iXon DU-885KCSO-VP, Oxford instruments) collected images through a quad-band dichroic mirror and emission filter set (TRF89901-EM-ET-405/488/561/640, Chroma Technology, Bellows Falls, VT USA). The fluorescence measurements were carried out on RBC smears prepared on coverslips (nominal thickness 150 $\upmu$m).

### Preprocessing of the microscopy images

To detect single cells in RBC images, the contrast of the images was increased. In case of the AFM images, Otsu’s method^49^ was used to calculated the thresholding value for segmentation. The binary mask was then handled by the Hough gradient method^44,45^, implemented in opencv to detect single cells. The light and fluorescence microscopy images were treated by increasing brightness, contrast, colour, and sharpness in case of low contrast images^50^ (PIL image enhance). The cell detection was controlled by five parameters (Table 4), where mDist describes the minimum distance between circle centres, par1 is a measure of how strong the edges of the circles need to be, par2 sets how many edge points the algorithm has to find to declare the object to be a circles, and minR and maxR set the minimum and maximum size of the circle.

**Table 4:** Set of values used for the Hough gradient method with the respective microscopy technique.

Imaging method	mDist	par1	par2	minR	maxR
AFM	28	10	8	14	30
Light microscopy/fluorescence microscopy	28	20	18	14	30

### Architecture of the neural network

For the classification of the cell stages, a simple convolutional, python-based neural network (CNN) was used. The input was either a two-dimensional image, four, two or one one-dimensional cross-section. Prior to training, the two-dimensional images were scaled and the cross-sections were normalised to represent the relative progression of the intensity and height profile. The data set was then labelled with the respective stage. The algorithm was implemented in Python 3.7, including *tensorflow*^51^ with keras for the NN.

Due to the measurement technique, the samples were imbalanced. To equalise the number images for each category, the data was augmented by rotation, scaling, and reflection in case of the two-dimensional images and oversampled by randomly duplicating examples from the minority classes and adding them to the data to create a balanced set. For the AFM images, an additional augmentation was performed to increase the overall amount of data.

**Figure 9:**
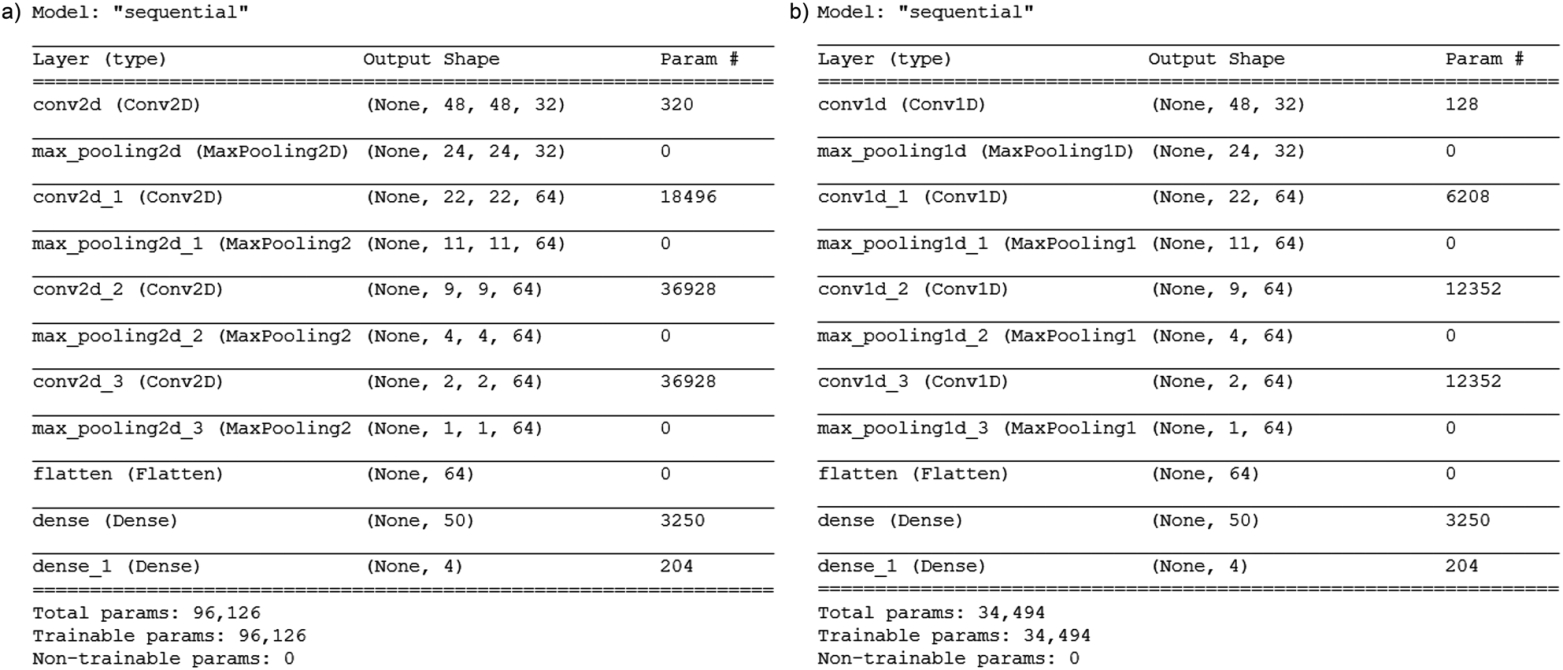
Model architecture of the neural network for 2D microscopy images (**a**) and two orthogonal 1D cross-sections (**b**).

The model architecture of the neural network used for the classification of the two- and one-dimensional images of RBCs is shown in Fig. 9. For the analysis of the two-dimensional images, we created a network (Fig. 9a) consisting of a single two-dimensional convolutional layer with 32 feature maps of kernel size 3, and three two-dimensional convolutional layers with 64 feature maps of kernel size 3. Each was followed by pooling layers to reduce the size of the input. The network was completed by two fully connected dense layers with 50 and 4 units with the “softmax“ activation function for the last layer and the “relu“ activation function for the other layers. In total, the classifier trained on 96,126 parameters in the two-dimensional case. To reduce overfitting, we used a kernel regulariser (l2(0.001)) in the first dense layer. We specified the loss function to “categorical_crossentropy“ to evaluate our set of weights and the optimizer “rmsprop“. Then, we fitted the model on our training set with a batch size of 20, running for 20 epochs. Our data was split into 75% training, 15% validation and 10% test set. To classify the characteristic cuts, we used the same architecture, but one-dimensional convolutional layers, leading to a classifier with a total number of 34494 trainable parameters.

## Data Availability

All data used for the analysis described in this manuscript are freely available and have been deposited in an online repository (Characteristic cuts and NNs: https://github.com/Reducing-data-dimension-boosts-neural-network-based-stage-specific-malaria-detection; Training, validation, and test set: https://zenodo.org/record/6866337). The complete cell evaluation program is available at https://github.com/KatharinaPreissinger/malaria_stage_classifier. Details about the implementation can be read on https://malaria-stage-classifier.readthedocs.io/en/latest/index.html.
